# ETHNICITY IN RESEARCH ON OLDER MIGRANTS: HOW THE SCHOLARSHIP CONCEPTUALIZES IT AND WHERE WE NEED TO HEAD

**DOI:** 10.1093/geroni/igad104.1633

**Published:** 2023-12-21

**Authors:** Sandra Torres

**Affiliations:** Uppsala University, Uppsala, Uppsala Lan, Sweden

## Abstract

A recently completed scoping review of peer-reviewed articles on older ethno-racialized minorities has shown that although conceptual and theoretical advancements have been made in the field of ethnicity and race, the social gerontological literature on these minorities continues to regard ethnicity in essentialist ways, conflates ethnicity with race and seldom operationalizes what these social positions mean to aging and old age. The impact of ethnicity is, in other words, almost always inferred in this research, which is why the field has been deemed to be characterized by data-richness and theory-poorness. Over the past three years, researchers working on the nexus of aging and migration have surveyed the current state of knowledge on older migrants (focusing on migrancy, rather than ethnicity and/ or race). One of these inventories is a meta-analysis of how research on older migrants conceptualizes ethnicity, and the manner in which this social position is presumed to impact on migrants’ old age- and aging-related experiences. At stake has also been the assessment of whether this literature differs from the scholarship on older ethno-racialized minorities, and the ways in which that scholarship conceptualizes ethnicity. This presentation offers insight into what this inventory has found out. In focus are the current state of affairs about how ethnicity is approached in research on older migrants, and the launching of a theoretically-informed and empirically-astute research agenda for the study of ethnicity in gerontology.

